# Genome-wide association study on chronic postsurgical pain in the UK Biobank

**DOI:** 10.1016/j.bja.2024.12.008

**Published:** 2025-01-25

**Authors:** Song Li, Masja K. Toneman, Luda Diatchenko, Marc Parisien, Kris C.P. Vissers, Richard P.G. ten Broek, Regina L.M. van Boekel, Marieke J.H. Coenen

**Affiliations:** 1 Department of Human Genetics, Radboud Institute for Health Sciences, Radboud University Medical Center, Nijmegen, the Netherlands; 2 Department of Surgery, Radboud Institute for Health Sciences, Radboud University Medical Center, Nijmegen, the Netherlands; 3 Faculty of Dental Medicine and Oral Health Sciences, Department of Anesthesia, Faculty of Medicine, Alan Edwards Centre for Research on Pain, McGill University, Montreal, QC, Canada; 4 Department of Anesthesiology, Pain and Palliative Medicine, Radboud Institute for Health Sciences, Radboud University Medical Center, Nijmegen, the Netherlands; 5 Research Department Emergency and Critical Care, HAN University of Applied Sciences, School of Health Studies, Nijmegen, the Netherlands; 6 Department of Clinical Chemistry, Erasmus Medical Center, Rotterdam, the Netherlands

**Keywords:** chronic postsurgical pain, genetics, genome-wide association study, *GLRA3*, glycine receptor, risk prediction, UK Biobank

## Abstract

**Background:**

Chronic postsurgical pain (CPSP) persists beyond the expected healing period after surgery, imposing a substantial burden on overall patient well-being. Unfortunately, CPSP often remains underdiagnosed and undertreated. To better understand the mechanism of CPSP development, we aimed to identify genetic variants associated with CPSP.

**Methods:**

A genome-wide association study was conducted in a cohort of 95,931 individuals from the UK Biobank who had undergone different surgical procedures. Three analyses were performed: (1) case–control analysis (2923 cases with CPSP and 93,008 controls), (2) ordinal analysis in three groups based on time of analgesics use (*n*=95,931), and (3) a meta-analysis combining our dataset with a recent publication (*n*=97,281).

**Results:**

In the case–control analysis, one genetic locus within *GLRA3* displayed a genome-wide significant (*P*<2.5×10^−8^) association with CPSP, and nine loci displayed suggestively significant associations (*P*<1×10^−6^). The ordinal analysis aligned with the case–control analysis, with an additional locus (rs140330443) reaching genome-wide significance. In the meta-analysis with the recently published dataset, the single nucleotide polymorphism (SNP) rs17298280 in the *GLRA3* gene remained significant (*P*=2.19×10^−9^).

**Conclusions:**

This study contributes new insights into the genetic factors associated with CPSP. The top hit *GLRA3* is known for involvement in prostaglandin E2-induced pain processing pathways. Our study provides a foundation for future investigations into the function of these risk variants and the mechanisms underlying CPSP by offering summary statistics. However, further validation in other cohorts is required to confirm these findings.

**Editor's key points**

• Approximately 23 million people experience chronic postsurgical pain (CPSP) every year.
• Identification of genetic variants associated with CPSP should enhance the prediction and management of CPSP.
• In the present study based on data from the UK Biobank, the authors identified one genome-wide significant locus (*GLRA3*) associated with chronic postsurgical pain. The latter encodes a protein in the glycine receptor family, which plays an important role in chronic inflammatory pain.
• This study adds to our knowledge of the genetic factors associated with CPSP.
• Further validation in other cohorts is required to confirm these findings.

Approximately 23 million individuals experience chronic postsurgical pain (CPSP) annually.^1^ CPSP has been linked to a reduced overall quality of life, placing a substantial emotional and physical burden on patients.^2^

The occurrence of CPSP (5–85%) depends on the surgical site, type of surgery, likelihood of nerve damage, and perioperative factors.^3^ CPSP is still underdiagnosed and undertreated.^4^ The management of CPSP might be improved by using individualised risk prediction for clinical decision-making.^5^ Although several identified CPSP risk factors have been included in risk prediction models,^6^^,^^7^ adequate prediction in clinical practice has not been achieved.^8^

The accuracy of CPSP prediction could be enhanced by incorporating genetic factors into prediction models. However, identifying genetic factors remains challenging.^9^ Two recent systematic reviews on genetic association studies of (chronic) postsurgical pain showed that only three variants (*OPRM1* rs1799971, *COMT* rs4680, and *KCNS1* rs734784) remained significantly associated with CPSP after meta-analysis.^10^^,^^11^ In addition, the focus of genetic association studies has been on acute pain (such as analgesic requirements and pain score after surgeries^12^, ^13^, ^14^), and most previous studies are candidate gene studies, which might overlook the beyond-known mechanisms. Only three genome-wide association studies (GWASes) on CPSP have been published in relatively small cohorts (a few hundred to 1700 subjects), showing inconsistent results.^12^^,^^14^^,^^15^ However, hypothesis-free methods (such as GWAS) in large cohorts are needed to discover the genetic background of CPSP further. Furthermore, polygenic risk scores (PRSs) based on GWASes have the potential to explain more phenotypic variance compared with single variant tests. Incorporating PRSs in prediction models for chronic (postsurgical) pain might improve predictive accuracy.^4^

This paper aims to identify genetic variants associated with CPSP in selected surgeries using data from the UK Biobank (UKB) through a GWAS. The exploratory goals were to investigate shared genetic factors and genetic correlations among CPSP development in different surgeries and other phenotypically related traits reported in previous research.

## Methods

**Fig 1:**
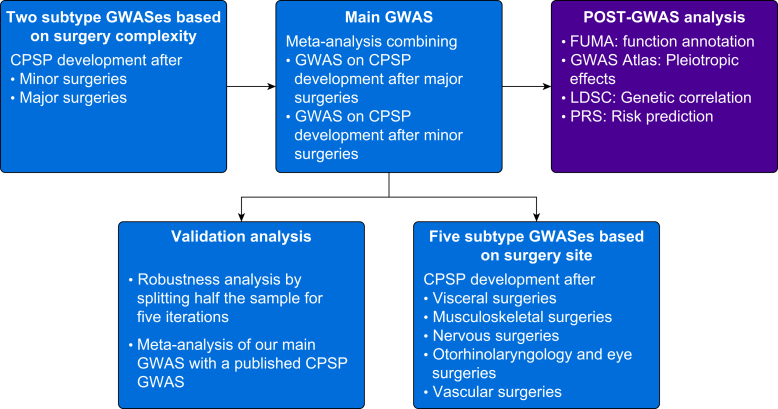
Analysis overview. CPSP, chronic postsurgical pain; FUMA, an integrative web-based platform for functional mapping and annotation of genome-wide association studies; GWAS, genome-wide association study; LDSC, linkage disequilibrium score regression; PRS, polygenic risk score; UKB, UK Biobank. The background color dark blue indicates genome-wide association analyses or its validation analyses, while the color dark purple indicates post-genome-wide association functional analyses.

We performed a meta-analysis GWAS for CPSP development (using a binary outcome) after major and minor surgeries, referred to as the main analysis in the following text. The results were carried forward for post-GWAS analyses. Figure 1 depicts the study workflow. This study was pre-registered on the Open Science Framework (https://osf.io/h6cr9/).

### Study cohorts

UKB data were used for the analyses. A description of the cohort can be found in the Supplementary methods.

### Inclusion and exclusion criteria

**Fig 2:**
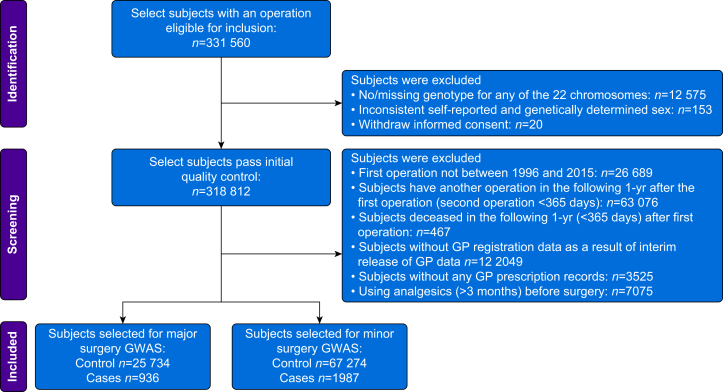
Flowchart of patient selection for chronic postsurgical pain. GWAS, genome-wide association study.

Subjects were considered eligible for inclusion if their initial surgery records were dated between 1997 and 2015. Only initial surgeries were selected for analysis as prior surgical procedures may affect CPSP development. Supplementary Table S1 lists included surgeries, surgery complexity, and corresponding OPCS4 codes. After quality control (QC) procedures (Fig. 2), subjects were divided into two groups (i.e. major and minor surgeries) based on the complexity of the surgeries.

Subjects were excluded if they met the following criteria: (1) failing routine GWAS sample QC (Supplementary methods); (2) withdrawing informed consent; (3) surgery records falling outside the established date range; (4) undergoing another surgery within the 1-yr follow-up period after their initial surgery; (5) deceased during follow-up; (6) without general practitioner (GP) registration data (a release of GP data was used, which covered approximately 45% of all UKB participants, resulting in the exclusion of 122,049 participants); (7) without any prescription records in the GP data; and (8) pre-existing chronic pain as suggested by more than 3 months of analgesic records within the year before surgery.

### Chronic postsurgical pain phenotype definition

Postoperative pain was determined based on the moment of the surgery and prescription records. After the initial surgery, 1-yr analgesic prescription records were extracted following the surgery event, primarily encompassing NSAIDs and opioids (see Supplementary Table S2 for included drugs). This follow-up period of 12 months was divided into intervals of 30 days, and the presence of analgesic prescription records within a specific interval denoted pain treatment during that particular month.

Based on the duration of analgesic prescription, patients were dichotomised into two groups: cases, defined as those with a minimum of 4 months (consecutive or non-consecutive) of analgesic consumption, and controls, comprising individuals with 3 or fewer months of analgesic use. Sample sizes and the number of cases and controls for all GWASes can be found in Supplementary Table S3.

Given that a complete recovery from major surgery can extend beyond 6 months,^16^ an ordinal score ranging from 1 to 3 was used. Score 1 indicates analgesics use for 3 months or less; score 2 indicates analgesics use between 3 and 6 months; and score 3 indicates analgesic use exceeding 6 months after their surgical procedure (see Supplementary materials).

### Genome-wide association analyses

We conducted two GWASes for CPSP development after major or minor surgery in the UKB. The main analysis was performed with the binary phenotype (cases with CPSP and controls without CPSP) combining results from major and minor surgeries in a meta-analysis. Major and minor surgeries were not directly combined in one analysis, as the genetic background between these two might be different. Next, we performed an analysis based on the ordinal phenotype (see above) as an exploratory analysis. For validation purposes, a meta-analysis combining our GWAS meta-analysis with a published paper^14^ on CPSP GWAS meta-analysis was performed. In the published study, patients undergoing hysterectomy, mastectomy, abdominal surgery, hernia repair, or knee surgery were recruited and genotyped, and postsurgical pain was assessed 3–6 months on a numeric rating scale (0–10 scale) after surgery.

Statistical power was calculated for the main GWAS using the following parameters: 2923 cases and 93,008 controls, a significance level of 2.5×10^−8^ (corrected for multiple testing, the same test for binary and ordinal phenotypes), a CPSP prevalence of 0.03 (based on the observed prevalence in the participants included in the analysis), a minimum effect allele frequency of 0.1, and a minimum effect allele relative risk of 1.3.

Seven subtype GWASes were performed, including two based on surgery complexity (CPSP development after either major or minor surgeries) and five GWASes based on different surgery site. Details of the GWAS meta-analysis, subtype GWASes, and the post-GWAS analyses can be found in the Supplementary methods. The number of selected genetic principal components was based on a scree plot (Supplementary Fig. S1).

### Validation of significant loci

For GWAS-identified loci, the lead SNPs are the most statistically significant SNPs within each locus. Independent SNPs are those in linkage disequilibrium (LD, with *r*^2^>0.6) with the lead SNP and remain statistically significant after conditioning on the lead SNPs. A robustness analysis for suggestively significant loci (*P*<1×10^−6^) was performed by splitting the main GWAS datasets into two equally sized subsets five times and then comparing the single variant results within these subsets for validation.

To determine whether the identified loci in this study were also reported in a published GWAS on CPSP, candidate SNPs (in LD, *r*^2^>0.6) with suggestively significant independent SNPs in our study were examined in that study.^14^ In addition, to compare our results with the published study on CPSP, we used PRSs to investigate whether our GWAS meta-analysis results can predict CPSP development in their study.

To validate previously published results on CPSP, we checked *P*-values for three variants that showed statistically significant association with CPSP in two systematic reviews^10^^,^^11^ in our dataset. In addition, significant results from two earlier published GWAS studies^12^^,^^14^ were checked. In the Introduction, we described three previously published GWASes. We did not include the analysis of van Reij and colleagues^15^ as this dataset was also included in the paper of Parisien and colleagues.^14^

### Risk prediction by polygenic risk score

To predict an individual's genetic predisposition to CPSP development across major and minor surgeries, we constructed regression models using GWAS results after major surgeries to predict CPSP development after minor surgeries. Typically, a PRS is built from the GWAS results, where an individual's genetic risk is the sum of all their risk alleles weighted by the significance of the corresponding allele.^17^ Two models were built: the null model, which included only GWAS covariates as predictive factors, and the PRS model, which incorporated PRS and GWAS covariates. The variance explained by the PRS model subtracted from the variance explained by the null model is the variance explained by the PRS. The PRSs were generated using the LDpred2 algorithm with the ‘auto’ option.^18^ This option facilitates direct estimation of model parameters from available data without external training data.

The same approach was applied to predict the risk of self-reported CPSP (Data-Field ID: 120005) within the UKB. To avoid sample overlap, individuals included in the GWAS were excluded from subjects with self-reported CPSP.

## Results

### Patient characteristics and phenotype validation

After QC procedures, we identified 26,670 subjects with major surgeries and 69,261 subjects with minor surgeries within the UKB dataset (Fig. 2). Patient characteristics of the combined dataset (major and minor surgeries) are presented in Table 1. Pre-existing pain before surgery was similar between cases and controls, indicated by prescription record numbers before surgery. In line with our selection criteria, cases consumed significantly more analgesics than controls after surgery (*P*<0.0001; Supplementary Fig. S2).

**Table 1:** Characteristics of participants without CPSP (control) and with CPSP (cases) after selected surgeries in the UK Biobank. Age and BMI are presented as mean (sd). Types of surgery are presented as the count and percentage of controls or cases in each surgery types. Opioid user is presented as the count and percentage of opioid users in cases and controls, respectively. The opioid/analgesic prescription number is present as median (sd) as the distribution is highly skewed. CPSP, chronic postsurgical pain; sd, standard deviation. ∗Subjects with more than one surgery subtype are classified as multiple sites. ^†^The median of opioid prescription number is calculated based on participants that used opioids (opioid users).

	Controls (*n*=93,008)	Cases (*n*=2923)	*P*-value
Females, *n* (%)	52,228 (56.15)	1495 (51.15)	<0.0001
Age(yr), mean (ranges)	52.6 (22, 78)	57.6 (29, 77)	<0.0001
BMI (kg m^−2^), mean (sd)	27.48 (4.73)	28.71 (5.05)	<0.0001
Types of surgery, *n* (%)			<0.0001
Visceral	61,267 (97.57)	1529 (2.43)	
Musculoskeletal	14,244 (96.00)	593 (4.00)	
Otorhinolaryngology and eye	8120 (98.02)	164 (1.98)	
Nerves	3116 (93.86)	204 (6.14)	
Multiple sites∗	3238 (92.49)	263 (7.51)	
Vascular	3023 (94.68)	170 (5.32)	
Opioid user, *n* (%)	44,325 (47.66)	2418 (82.72)	
Opioid prescription number, median (sd)^†^	3 (28.32)	12 (63.29)	
Analgesic prescription numbers, median (sd)	0 (0.71)	7 (4.76)	
Analgesic prescription months, median (sd)	0 (0.60)	5 (1.84)	

All tested covariates were significantly different between cases and controls and are incorporated as covariates in the GWAS. In addition, the median surgery record numbers (Supplementary Fig. S2A) were similar between cases and controls, indicating no potential influence of surgery complexity on our phenotype definition. In addition to our main GWAS analysis combining major and minor surgeries in a meta-analysis, an ordinal analysis, and an analysis combining the data with a recent publication, we also performed several sub-type analyses.

### Genome-wide association analysis

In the meta-analysis combining the GWAS after major and minor surgeries (main GWAS), no inflation was observed in the results (Supplementary Fig. S3C). A single locus on chromosome 4 reached genome-wide significance (Supplementary Fig. S4C). Within this locus, the most significant SNP was rs17298280 in the intronic region of the *GLRA3* gene (*P*=2.17×10^−9^). The minor allele (G) frequency in the cases and controls were 0.163 and 0.193, respectively. Within the same locus, an additional independent SNP remained statistically significant after conditioning on the lead SNP. Table 2 summarises the lead SNPs within each locus that surpassed the suggestively significant threshold (*P*<1×10^6^). Ordinal GWAS results were consistent with the GWAS meta-analysis (Supplementary Table S4 and Fig. S5), with an additional locus (rs140330443 within *CRAMP1L*) reaching genome-wide significance.

**Table 2:** Overview of the lead SNPs passing the suggestive significance level in the GWAS meta-analysis on chronic postsurgical pain. Bold font indicates the SNP that passed the genome-wide significant threshold (2.5×10^−8^). BETA (se), effect size and standard error; CHR:POS, coordination of the SNP; GWAS, genome-wide association study; HetPVal, heterogeneity *P*-value; MAF, minor allele frequency (obtained in FUMA based on the 1000 Genomes Project Phase 3 data).

SNP	CHR:POS	Effect allele	MAF	BETA (se)	Location	Nearest_Gene	Direction	*P*	HetPVal	Major GWAS *P*	Minor GWAS *P*
**rs17298280**	**4:175634898**	**C**	**0.207**	**0.0059 (0.0010)**	**Intronic**	***GLRA3***	**++**	**2.17E–09**	**2.63E–01**	**8.10E–05**	**2.86E–06**
rs184832856	2:139555831	A	0.006	–0.0256 (0.0049)	Intergenic	*NXPH2*	– –	1.64E–07	5.55E–01	2.93E–02	1.35E–06
rs140330443	16:1672582	A	0.005	0.0240 (0.0046)	Intronic	*CRAMP1L*	++	1.75E–07	5.01E–03	3.49E–07	2.06E–03
rs186819635	1:95071179	A	0.003	0.0252 (0.0049)	Intergenic	*RP11-86H7.6*	++	2.70E–07	2.74E–01	1.09E–01	4.63E–07
rs12143186	1:68737833	T	0.111	–0.0062 (0.0012)	Intergenic	*COX6B1P7*	– –	3.20E–07	3.88E–02	1.43E–05	5.84E–04
rs10032594	4:175645417	C	0.335	–0.0042 (0.0008)	Intronic	*GLRA3*	– –	4.08E–07	8.47E–01	1.90E–02	6.01E–06
rs145636748	1:95059856	A	0.008	0.0197 (0.0039)	Intergenic	*RP11-86H7.6*	++	5.46E–07	8.25E–01	8.57E–03	1.69E–05
rs117130005	17:19604614	T	0.017	0.0173 (0.0035)	Intronic	*SLC47A2*	++	6.26E–07	5.16E–01	2.63E–03	4.94E–05
rs2160419	13:72314346	A	0.014	–0.0182 (0.0037)	Intronic	*DACH1*	– –	7.70E–07	9.05E–01	1.66E–02	1.32E–05
rs138470454	10:14101741	C	0.007	0.0247 (0.0050)	Intronic	*FRMD4A*	++	9.75E–07	6.02E–01	4.59E–02	5.82E–06

In the meta-analysis encompassing GWAS after major surgeries, GWAS after minor surgeries, and the published CPSP study,^14^ among all suggestively significant SNPs, only two SNPs (rs17298280 and rs12143186) were genotyped in our subjects and all cohorts from the published CPSP study. The SNP rs17298280 remained the most significant locus (*P*=2.19×10^−9^; Supplementary Fig. S6). The C allele of this SNP was positively associated with CPSP in the GWAS after major surgeries, GWAS after minor surgeries, and two cohorts from the published CPSP study. However, it showed a negative association in the remaining cohorts of the published study. Lead SNPs reaching suggestive significance in this meta-analysis are presented in Supplementary Table S5.

### Power and heritability analysis

The power for the meta-analysis of GWAS after major and minor surgeries was 0.693. The liability scale heritability for the GWAS after major surgeries was 0.1202 (SE 0.1170), and that for GWAS after minor surgeries was 0.0091 (SE 0.0617). However, the heritability for the main GWAS (combining the GWAS after major and minor surgeries) was unmeasurable (below zero).

### Functional annotation of SNPs

All variants in LD with the lead SNPs were noncoding variants (Supplementary Table S6). In the genome-wide significant locus (*GLRA3*), rs11133053 had the highest combined annotation-dependent depletion (CADD) score of 11.41. Within the same locus, two intronic SNPs (rs144351495 and rs17298280) were associated with active enhancers or promoters in multiple tissues, indicating potential regulatory functions. In addition, rs17298280 had a promising RegulomeDB score (2a) for a potential regulatory SNP.

The pleiotropic effects of lead SNPs in the meta-analysis were evaluated in the GWAS Atlas and GWAS Catalog. A list of associated traits that passed multiple testing thresholds is provided in Supplementary Tables S7 and S8. In the GWAS Atlas, 79 traits surpassed the significance threshold, with a focus on psychiatric and neurological traits. Notably, depression emerged as the most significantly associated trait with the lead SNP rs140330443 (*P*=4.26×10^−8^). Other phenotypically correlated traits, such as posttraumatic stress disorder, alcoholic drinks, BMI, and sitting height, were identified. Furthermore, in the GWAS Catalog, red blood cell count was identified as an associated trait for lead SNP rs12143186.

### Gene mapping and gene-based analysis

After mapping GWAS candidate SNPs to genes, we identified 42 genes (Table 3). Thirteen genes were mapped based on genomic location, six were identified through cis-expression quantitative trait locus (cis-eQTL) mapping, and 29 were annotated by SNPs within 3D chromatin interaction regions. Eight genes were identified through at least two of these mapping strategies. Fourteen genes were related to pain or relevant neurological functions, based on PubMed or Genecards.

**Table 3:** Genes mapped by SNPs in LD (*r*^2^>0.6) with lead SNPs in FUMA. ciMap, ‘Yes’ indicates chromatin interaction mapping; eqtlMapminP, the minimum eQTL *P*-value of mapped SNPs; qtlMapminQ, the minimum eQTL FDR of mapped SNPs; eqtlMapSNPs, the number of eQTL mapping SNPs to this gene; LD, linkage disequilibrium; minGwasP, the minimum *P*-value of mapped SNPs; posMapSNPs, the number of positional mapping SNPs to this gene. ^∗^Neurological function-related gene. ^#^Pain-related gene.

Lead SNPs	Gene symbol	chr	Methods used for gene mapping					
			posMapSNPs	eqtlMapSNPs	eqtlMapminP	eqtlMapminQ	ciMap	minGwasP
rs17298280	*ADAM29*	4	0	NA	NA	NA	Yes	2.55E–09
	*GLRA3 ^#^*	4	9	PsychENCODE_eQTLs	2.63E–04	1.94E–02	Yes	2.17E–09
rs117130005	*ALDH3A2^∗^*	17	0	NA	NA	NA	Yes	6.26E–07
	*SLC47A2*	17	2	NA	NA	NA	No	6.26E–07
	*ULK2^∗^*	17	1	GTEx/v8/Cells_Cultured_fibroblasts:GTEx/v8/Skin_Sun_Exposed_Lower_leg	2.01E–05	3.44E–10	No	2.60E–06
	*AKAP10^∗^*	17	0	eQTLGen_cis_eQTLs	4.13E–12	0	No	2.60E–06
	*LGALS9B*	17	0	eQTLGen_cis_eQTLs	8.55E–07	2.70E–03	No	4.76E–05
rs12143186	*WLS ^#^*	1	0	eQTLGen_cis_eQTLs	6.21E–14	0	No	1.71E–05
rs138470454	*FRMD4A^∗^*	10	1	NA	NA	NA	No	9.75E–07
	*HSPA14*	10	0	NA	NA	NA	Yes	9.75E–07
rs140330443	*CRAMP1L*	16	1	NA	NA	NA	No	1.75E–07
	*LA16c-431H6.6*	16	1	NA	NA	NA	No	1.75E–07
	*TSR3*	16	0	NA	NA	NA	Yes	NA
	*GNPTG*	16	0	NA	NA	NA	Yes	NA
	*HN1L*	16	0	NA	NA	NA	Yes	NA
	*MAPK8IP3^∗^*	16	0	NA	NA	NA	Yes	NA
	*HAGH*	16	1	NA	NA	NA	No	NA
	*FAHD1*	16	2	NA	NA	NA	No	NA
	*MEIOB*	16	2	NA	NA	NA	Yes	NA
	*HS3ST6 ^#^*	16	0	NA	NA	NA	Yes	NA
	*MSRB1^∗^*	16	0	NA	NA	NA	Yes	NA
	*RPL3L*	16	0	NA	NA	NA	Yes	NA
	*NDUFB10*	16	0	NA	NA	NA	Yes	NA
	*RPS2*	16	0	NA	NA	NA	Yes	NA
	*RNF151*	16	0	NA	NA	NA	Yes	NA
	*TBL3*	16	0	NA	NA	NA	Yes	NA
	*NOXO1*	16	0	NA	NA	NA	Yes	NA
	*AC005606.1*	16	0	NA	NA	NA	Yes	NA
	*GFER*	16	0	NA	NA	NA	Yes	NA
	*SYNGR3*	16	0	NA	NA	NA	Yes	NA
	*NTN3 ^#^*	16	0	NA	NA	NA	Yes	NA
	*TBC1D24^∗^*	16	0	NA	NA	NA	Yes	NA
	*PRSS33*	16	0	NA	NA	NA	Yes	NA
rs145636748; rs145636748:rs186819635	*F3^∗^*	1	1	NA	NA	NA	Yes	2.70E–07
rs184832856	*TMED5*	1	0	NA	NA	NA	Yes	1.70E–03
	*CCDC18*	1	0	NA	NA	NA	Yes	1.70E–03
	*ARHGAP29*	1	1	NA	NA	NA	No	1.70E–03
	*TMEM56*	1	0	NA	NA	NA	Yes	1.70E–03
	*RWDD3 ^#^*	1	0	NA	NA	NA	Yes	1.70E–03
rs186819635; rs145636748	*ABCD3*	1	1	eQTLGen_cis_eQTLs	2.38E–08	1.15E–04	No	1.51E–04
rs184832856	*HNMT ^#^*	2	0	NA	NA	NA	Yes	1.64E–07
rs2160419	*DACH1*	13	3	NA	NA	NA	No	7.70E–07

Gene-based association analysis in MAGMA (Generalized Gene-Set Analysis of GWAS Data | PLOS Computational Biology. https://journals.plos.org/ploscompbiol/article?id=10.1371/journal.pcbi.1004219) was performed for 19,296 genes (Supplementary Table S9). However, no statistically significant associations were identified (Bonferroni-corrected P-value 2.59 x 10^−6^).

### Validation analysis

In our robustness analysis, eight of the 10 variants in the meta-analysis successfully passed the validation with nominal significance (*P*<0.05) in all five iterations (Supplementary Table S10).

Candidate SNPs from our meta-analysis including major and minor surgeries were checked in the published CPSP study.^14^ None of these SNPs passed the Bonferroni-corrected *P*-value threshold (0.05/31). One SNP (rs62066262) passed the nominal significance threshold but was only genotyped in one cohort (Supplementary Table S11).

In the validation analysis using meta GWAS results to predict CPSP in the published CPSP study based on a PRS, the variance explained by the null model (with all GWAS covariates) was 8.23%. After introducing the PRS into the model, the explained variance increased only by 0.000144%. Using only the results from the GWAS after major surgeries for the PRS validation analysis produced comparable findings (data not shown).

Three SNPs reported to be associated with chronic pain from two published systematic reviews were checked in our dataset, and none of these three SNPs showed nominal statistical significance (Supplementary Table S12). Genome-wide significant variants identified by previously performed GWASes were checked as well. Only one of the four variants (rs114837251, near *MAP9*/*GUCY1A1*/*GUCY1B1*) showed nominal significance in our study (Supplementary Table S13).

### Risk prediction

In the PRS model using the results from the GWAS after major surgeries to predict CPSP development after minor surgeries, the null model (including only GWAS covariates) explained 9.85% of the variance. Upon introducing the PRS, there was a negligible increase of 0.0024% in explained variance.

In the PRS model using the results from the meta-analysis GWAS to predict self-reported CPSP in the UKB, the null model accounted for 0.3235% of the variance, and the PRS contributed to a slight increase of 0.01865% in explained variance. We conducted the same analysis using the results from the GWAS after major surgeries, yielding comparable results (data not shown).

### Subtype genome-wide association studies and genetic correlations

Subtype GWASes were performed based on surgery complexity and surgery site, and results can be found in Supplementary Figures S4 and S7 and Supplementary Tables S14 and S15.

CPSP development after different subtype surgeries showed nonsignificant moderate correlation coefficients. The same trend was observed for other phenotypically correlated traits (Supplementary Tables S16 and S17 and Fig. S8). More information on these two analyses can be found in the Supplementary materials.

## Discussion

We identified one genome-wide significant locus (*GLRA3*) associated with CPSP using the UKB in the meta-analysis of major and minor surgeries. This SNP remains genome-wide significant in the meta-analysis, which integrates data from our genome-wide association results (both major and minor surgeries) and a published CPSP study.^14^

The genome-wide significant locus is mapped to the *GLRA3* gene, encoding a protein (GlyRα3) of the glycine receptor subfamily, which are widely distributed throughout the central nervous system. GlyRα3 plays an important role in the downregulation of neuronal excitability and contributes to the generation of inhibitory postsynaptic current. The role of GlyRα3 varies depending on the nature of the pain. In an inflammatory pain mouse model, elevated COX2 led to the spinal release of prostaglandin E2, which inactivated GlyRα3 via phosphorylation. This GlyRα3-mediated inactivation of inhibitory neurones contributes to the central mechanisms of chronic inflammatory pain.^19^^,^^20^ In contrast, GlyRα3 appears to have little or no role in several neuropathic pain models.^21^^,^^22^ For instance, in partial sciatic nerve ligation model, Glra3^–/–^ mice show normal pain behaviour, mechanical allodynia, and thermal sensitivities.^20^ This all suggests that inflammatory pain might be an important underlying mechanism of CPSP.

Genes identified through suggestively significant loci may be involved in pain-related processes, as evidenced by gene expression studies. Notable examples include WLS, which exhibits high expression levels in chronic neuropathic pain patients^23^; RWDD3, which is downregulated after sciatic nerve ligation^24^; Netrin-3 (NTN3), whose reduced expression is strongly associated with the severity of diabetic neuropathic pain in a diabetic mouse model^25^; and HNMT which has been linked to the morphine dosage requirements in cancer pain patients.^26^ However, transcriptome-wide association analysis to find gene expression associated with CPSP by using S-PrediXcan in specific tissues showed no significant association (after Bonferroni correction) for the genes identified (data not shown). Furthermore, although some other genes are not directly implicated in pain, they are associated with other neurological functions that might contribute to pain development. For instance, HS3ST6 is involved in hereditary angioedema, characterised by acute episodic cutaneous or submucosal angioedema, often accompanied by abdominal pain.^27^

In the validation analysis of our identified SNPs, only one SNP (rs62066262) was replicated in a previously published study on CPSP^14^ with nominal significance. In the validation of previously identified SNPs in our study, only one SNP (rs114837251) approached the nominal significance threshold (*P*=0.05059) in our meta-analysis. It is important to note that our study is not a perfect replication of the previous study and vice versa, given several key distinctions. First, many SNPs in our study were not genotyped in their study, such as rs2160419, which was only genotyped in one out of six cohorts. Moreover, the phenotype definition was different between the two studies. In the published study, CPSP was assessed directly via a numeric rating scale or focusing acute pain within 48 h after surgery, whereas our study used a surrogate pain phenotype based on analgesic consumption. However, the previously published study emphasised the potential role of the adaptive immune system in CPSP development, aligning with our findings that implicate inflammatory pain as a significant component of CPSP.

The pleiotropic effects analysis of the lead SNPs reveals traits known to be linked with CPSP development, such as psychiatric traits (depression, posttraumatic stress disorder, and anxiety) and BMI.^7^^,^^28^ In addition, the GWAS Catalog indicates SNPs with pleiotropic effects of red blood cell count. This connection between red blood cell counts and pain is intriguing as red cell distribution width has been linked to chronicity in nonspecific low back pain.^29^

The low estimated SNP heritability in our study reflects the well-known ‘missing heritability’ problem in GWASes. This phenomenon can be attributed to SNP-based heritability calculations based on a subset of common genetic variants. As a result, the heritability estimation tends to be lower than those observed in twin studies. Several factors contribute to this disparity, including that the genotyped SNPs in GWASes are not in complete LD with the causal variants; nonadditive effects, rare variants, and structural variants often are undetected in SNP-based heritability assessments.

Although PRS analysis in our study lacks power owing to insignificant GWAS results, we should not overlook the potential of using the PRS as a potential predictor of CPSP.^30^, ^31^, ^32^ There is a growing movement to incorporate the PRS in risk prediction for clinical care.^33^^,^^34^ In the context of CPSP, risk prediction is in its initial stages. Two studies explored building a PRS model for CPSP.^4^^,^^35^ The models that integrate the PRS into risk assessment exhibit a higher predictive accuracy for CPSP than non-genetic models.^4^ To replicate and validate these findings, further studies need to include large-scale cohorts to construct robust PRS models, which underscore the need for large-scale GWAS efforts. An example of such an initiative is the PPG cohort at our research centre.^36^

One of the strengths of our study is the inclusion of different types of surgeries, leading to a substantial sample size. This large sample size allowed us to investigate the common genetic underpinnings of CPSP across various surgical procedures. However, it is crucial to acknowledge the limitations of our study. Instead of directly measuring CPSP, we used a proxy method relying on analgesic prescription records to define chronic pain. The advantage of this phenotype definition is that it captures more severe pain symptoms necessitating medication, as indicated by the relatively low prevalence of CPSP in our study. We cross-checked our phenotype definition with the self-reported CPSP in the UKB (after removing subjects without prescriptions), as shown in Supplementary Table S18. The agreement between these two classifications was compared using unweighted Cohen's kappa (κ=0.02), which only shows slight agreement. Subjects included as controls in our analysis were indicated to suffer from CPSP in the self-reported data. This can be explained by two reasons. First, our definition did not include all surgeries and only considered the first surgery for analysis. Thus, subjects may develop CPSP from subsequent surgeries. Second, evidence indicates that there are patients experiencing significant pain but opting not to pursue treatment.^37^ It might happen that subjects with CPSP do not always seek prescriptions. Subjects without CPSP in the self-reported data but marked as cases in our phenotype definition can be explained by several factors: subjects having high pain tolerance, effective pain control by prescribed analgesics, and analgesics prescribed for conditions other than CPSP. In addition, as the UKB team mentioned, the chronicity and location of CPSP are not well documented in their questionnaire and should not be seen as the standard for examining CPSP. The team advised not to use this phenotype for GWASes. Therefore, we did not use this phenotype in our analysis but used analgesic consumption 1 yr after an operation as a phenotype. CPSP development could be a combination of genuine CPSP and a suboptimal response to analgesics. Despite these limitations, the substantial sample size included in our study potentially mitigates this issue. Another limitation is that our study lacks statistical power, as it did not meet the commonly accepted threshold of 0.8. Although our sample size is relatively large (*N*=95,931), complex traits require even larger sample sizes to fully elucidate the genetic architecture of such traits, as they are influenced by numerous common variants with very small effect sizes. For example, the estimated heritability of height in studies before 2010 was only 5%, much lower than the pedigree-based heritability estimate of 80%, despite those studies being considered large at the time (*n*=1000 to 10,000).^38^ A closer estimation of the pedigree-based heritability was achieved in a more recent GWAS meta-analysis with sample size exceeding 250,000 for height.^39^ Thus, although our sample size is substantial, it is still relatively small for precise heritability estimation.

Our study provides a foundation by offering summary statistics for future investigations into the function of these risk variants and the mechanisms underlying CPSP. In subsequent research, we advocate for conducting GWASes with substantial sample size and consistent phenotype definition, as this will be instrumental in advancing risk identification and tailoring personalised treatments for individuals at risk of CPSP.

## Authors’ contributions

Analysed the data and prepared the manuscript: SL

Provided the data for the PRS analysis: LD, MP

Contributed to the phenotype definition: RLMvB, MKT, RPGtB, KCPV

Conceptualised the study and supervised the overall project: MJHC

Reviewed and approved the final version of the manuscript: all authors

## Funding

China Scholarship Council (201908130179 to SL).

## Data availability statement

Summary statistics of the primary analysis are available at DANS archive (https://doi.org/10.17026/LS/486TJZ). Gene mapping results are available at FUMA (https://fuma.ctglab.nl/snp2gene/266351).

## Declaration of interest

The authors declare that they have no conflicts of interest.
