# Supplementary material for: Genome-wide association study on chronic postsurgical pain in the UK Biobank
Source: Br J Anaesth. 2025 Jan 25;134(3):783–92. doi: 10.1016/j.bja.2024.12.008 (PMC11867066; doi:10.1016/j.bja.2024.12.008)
Supplement: Multimedia component 1 [file mmc1.docx]

# Supplementary method

## Study cohorts

UK biobank is a prospective cohort comprising individuals recruited from the general population aged 40 to 69 across the United Kingdom (UK), as described in more detail elsewhere ^1^. The phenotype definition was based on the primary care (general practitioners, GP) data within UKB, a longitudinal dataset encompassing structured diagnoses and prescription information. Notably, at the time of our analysis (2023 July), the interim release of GP data covered approximately 45% of all UKB participants (all provided informed consent as part of the UKB).

## Note of phenotype definition

The surgery complexity information was sourced from the Clinical Coding and Schedule Development Group ^2^ and the clinician's expertise. In this study, surgeries categorized as major, xmajor, and complex were collectively classified as major, while minor and intermediate were grouped as minor (Table S1).

Notably, the phenotype definition did not completely follow the IASP CPSP definition criteria requiring consecutive prescription records. As the analgesic prescription records were used as a proxy to CPSP, this approach was not ideal to perfectly capture the continuous presence of pain because it depended on the total prescriptions received by patients and the frequency of medication delivery.

## Oridinal phenotype definition

Specifically, an ordinal score ranging from 1 to 3 was assigned: a score of 1 was assigned to individuals using analgesics for three months or less (n ≤ 3), a score of 2 denoted those using analgesics for a duration between three to six months (3 < n ≤ 6), and a score of 3 was assigned for those with analgesic use exceeding six months (n > 6) following their surgical procedure. The ordinal phenotype GWAS was conducted only in a single analysis that included both major and minor surgeries.

Heritability analysis

Liability-scale (SNP-based) heritability was calculated by using LDSC ^3^ with Europeans from the 1000 Genomes Project ^4^ as the LD reference panel. The major histocompatibility complex region (chr6: 26–34 Mb) was excluded. This calculation was based on several assumptions: a population prevalence of 0.10 for individuals who have undergone surgeries in general, a CPSP prevalence of 0.04 for subjects with major surgeries, and a CPSP prevalence of 0.03 for subjects with minor surgeries and in the meta-analysis.

GWAS Routine Sample and genotype QC

Subjects meeting the following criteria were included for analysis: Subjects with consistent self-reported and genetically determined sex, genetically determined white British ancestry, without putative sex-chromosome aneuploidy, not considered outliers due to missing heterozygosity, individual call rate > 90%, all relevant covariates are available.

Markers on autosomes that meet the following criteria were included: SNPs with an imputation quality score (INFO scores) of greater than 0.8, Minor Allele Frequency (MAF) > 0.005, Hardy-Weinberg equilibrium (HWE) test P > 10^-6^, Genotyping call rate > 95%.

Subtype Genome-wide association analyses

We conducted two subtype GWASes based on surgery complexity, i.e., GWAS on CPSP development after major or minor surgery. Both analyses followed the same analytical procedures using the linear mixed model function in GCTA ^5^. In GCTA, MLM-based tool (fastGWA) controls for population stratification by principal components and for relatedness by a sparse genetic relationship matrix ^5^. The same set of covariates applied to all GWASes models, including age at time of surgery, gender, assessment center, genotyping array type, the first five genetic principal components, and surgery types. The number of selected genetic principal components was based on a scree plot (Figure S1). Covariates were compared using t-tests for continuous variables and chi-square tests for categorical variables across different groups. Significance thresholds for GWAS were set at commonly accepted levels: P < 5 x 10^-8^ for genome-wide significance ^6^ and a suggestively significant threshold of 1 x 10^-6^ < P < 5 x 10^-8^ ^7^.

We conducted another five subtype GWASes based on surgery type, to explore the genetic correlations between CPSP development for various surgeries. These subtypes encompassed visceral surgeries, musculoskeletal surgeries, nervous surgeries, otorhinolaryngology and eye surgeries, and vascular surgeries, which could be major and minor surgeries. Notably, individuals who undergoned two different types of surgery on the same day (such as both visceral and musculoskeletal surgeries) will be included in our GWAS meta-analysis (see below) but excluded from this subtype-specific GWAS analysis. For this subtype-specific GWAS analysis, we focused on the binary phenotypes. These analyses followed the same analytical procedures as the subtype analyses based on surgery complexity mentioned above.

GWAS Meta-analysis

To explore if loci were potentially involved in CPSP development across major and minor surgeries, we conducted a meta-analysis using the fixed-effect inverse-variance weighted model in METAL, integrating the GWAS results on CPSP development after major and minor surgeries. Please note that there was not overlap for either cases or controls between GWAS on CPSP development after major surgeries and GWAS on CPSP development after minor surgeries as subjects were partitioned by their first major / minor surgery. The results of this meta-analysis will be used for subsequent post-GWAS analyses. The ordinal phenotype for the meta-analysis of GWAS on CPSP development after major or minor surgeries was analyzed with the ordinal regression in OrdinalGWAS ^8^. Significance thresholds for GWAS meta-analysis of CPSP after major or minor surgeries were set at: P < 1.7 x 10^-8^ for genome-wide significance to account for the multiple testing correction (for binary and ordinal phenotype, and the meta-analysis mentioned below).

Additionally, to further enhance the statistical power to identify novel loci associated with CPSP and validate our GWAS findings, another meta-analysis was performed encompassing our GWAS on CPSP development after major surgeries, GWAS on CPSP development after minor surgeries, and a previously published GWAS on CPSP ^9^. For the previously published CPSP study, summary statistics from the GWAS on binary CPSP outcomes were provided for each subcohort. We conducted a meta-analysis of CPSP after major surgery, CPSP after minor surgery, and the six subcohorts from the previously published study.

Functional annotation of SNPs

FUMA (Functional Mapping and Annotation of Genome-Wide Association Studies) was used to identify lead SNPs and significant independent SNPs, which are SNPs in linkage disequilibrium (LD, with r^2^ > 0.6) with the lead SNP and remain statistically significant after conditioning on the lead SNPs. To understand the SNPs’ functions and identify potential regulatory SNPs, all SNPs in LD (r^2^ > 0.6) with significant independent SNPs were annotated by Variant Effect Predictor, ANNOVAR, RegulomeDB (all within FUMA), and Haploreg.

Additionally, we explored potential pleiotropic effects associated with lead SNPs by querying the GWAS Catalog and GWAS Atlas. Traits that passed the Bonferroni correction threshold (0.05/10) in the original GWAS reported in the database are presented in the results section.

Gene mapping and gene-based analysis

SNPs in LD (r^2^ > 0.6) with lead SNPs were mapped to genes through three approaches in FUMA: positional mapping, cis-expression quantitative trait locus (cis-eQTL) mapping, and open chromatin mapping. In the positional mapping strategy, we assessed whether SNPs fall within a gene region (10 kilobases window). In the eQTL mapping, SNPs were searched in the database for association with gene expression levels. The significance threshold is FDR < 0.05 for cis-eQTL mapping. Open chromatin mapping can identify chromatin interactions based on prior spatial interaction data, even if these interactions occurred over considerable physical distances. The significance threshold in open chromatin mapping was set at FDR < 1x 10^-6^. Following the gene mapping process, we searched GeneCards and PubMed to investigate whether the candidate genes were functionally related to pain or neurological functions, providing further insights into their potential role in CPSP.

A gene-based analysis was conducted in MAGMA. SNPs were selected to map onto genes with a window size of 50 kb.Gene-based P-values were calculated based on the P-value of SNPs mapped to a specific gene. The significant threshold for gene analysis is set as 0.05/total number of genes included in the analysis (N=19296).

Genetic correlation

To investigate whether there are genetic confounders between phenotypic correlation with CPSP, genetic correlation analysis was performed: 1) between CPSP development after major and minor surgery, using GWAS on CPSP development after major surgeries and GWAS on CPSP development after minor surgeries results; 2) between CPSP development after different surgery types, utilizing the subtype-specific GWAS results mentioned earlier.

In addition, phenotypically correlated traits with CPSP were also investigated. A list of included traits can be found below. Given that the meta-analysis GWAS heritability result is unmeasurable, we utilized the GWAS on CPSP development after major surgeries results for genetic correlation with phenotypically correlated traits. The significance threshold for genetic correlation was set at 0.05 divided by the total number of tested correlations (0.05/15).

The genetic correlation of CPSP with other traits includes pain types experienced in the last month (headache, neck or shoulder pain, stomach or abdominal pain, hip pain, knee pain), ICD10 diagnosis of abdominal and pelvic pain (R10) as main or secondary diagnoses, ever vs. never smokers, and the use of opioids for personal consumption, which all are sourced from the GWAS Atlas. Additional traits from relevant papers, including back pain ^10^), neck/shoulder pain for 3+ months ^11^, knee pain for 3+ months ^11^, headaches for 3+ months ^11^, depression ^12^, and body mass index ^13^.

## Self-reported CPSP in UKB

The self-reported CPSP data was obtained from the UK Biobank's Experience of Pain questionnaire (Data-Field 120005). Participants were provided with response options including "Yes," "No," "Do not know," and "Prefer not to answer." It is important to note that the information collected through this questionnaire are insufficient to conduct Genome-Wide Association Studies (GWAS) related to pain. As per the UKB "It is essential to carefully record the duration, location, intensity and quality of pain as well as the temporal relationship to predisposing factors and co-morbidities (such as sleep, anxiety and depression). Whilst UK Biobank gathered data on pain during the baseline assessment, the level of phenotyping is not sufficient to undertake any pain-related GWAS.

# Supplementary results

Genome-wide association analysis

In the GWAS after major and minor surgeries analysis, no inflation was observed in the results, as evidenced by genomic control values of 1.00 and 1.01, respectively (Figure S3 for QQ-plot). No genome-wide significant hits were identified (P < 5 x 10-8) in either of these analyses (Figure S4A, Figure S4B). Lead SNPs surpassing the suggestively significant threshold are presented in Table S5 and Table S6 for surgery subtypes based on complexity or sites, respectively (see Figure S4 and S5 for Manhattan plots).

Genetic correlation

Genetic correlation analysis between the GWAS after major and minor surgeries was not feasible due to the low heritability observed in the GWAS after minor surgeries.

Genetic correlation analysis between the GWAS after major surgeries and other phenotypically correlated traits, showed strongest regression coefficient (rg) with the published CPSP study ^14^. Most chronic pain phenotypes displayed a positive genetic correlation, some exceptions are self-reported abdominal pain and abdominal pain as secondary diagnosis. Similarly, other phenotypically related traits, such as BMI and depression, also correlate positively. Important to note none of these genetic correlations reached statistical significance (Figure S7, Table S17).

No statistically significant genetic correlations were identified between subtype surgeries. However, rg values exhibited substantial magnitudes. Positive correlations were observed among musculoskeletal, vascular, and nerve subtypes, while negative correlations were noted with other surgery subtypes (Figure S7, Table S18).

# Supplementary figure legend

Figure S1: Percentage of variances explained by principal components in subjects with major (A) and minor (B) surgeries.

Figure S2: Number of operation codes (A), analgesic prescription numbers before (B) and after (C) surgeries in subjects included in the main GWAS on CPSP development after major and minor surgeries.

Figure S3: QQ plot of GWAS on chronic post-surgical pain in subjects undergone major (A), minor (B) and meta-analysis of major and minor surgeries (C).

Figure S4: Manhattan plot of genome-wide association analysis on chronic postsurgical pain. (A) GWAS on CPSP after selected major surgeries. (B) GWAS on CPSP after selected minor surgeries. (C) Meta analysis of GWAS on CPSP after selected major and minor surgeries. The red line corresponds to the genome-wide significance threshold of 5 × 10^-8^, whereas the blue indicates the suggestive threshold of 1 × 10^-6^. GWAS, genome-wide association analysis; CPSP, chronic postsurgical pain.

Figure S5: Manhattan plot of genome-wide association analysis on chronic postsurgical pain after selected major and minor surgeries using ordinal phenotype.

Figure S6: Meta-analysis of GWAS on CPSP development after major surgeries, GWAS on CPSP development after minor surgeries, and publish chronic postsurgical pain study.

Figure S7: Subtype GWASes in subjects undergone musculoskeletal (A), nervous (B), otorhinolaryngology and eye surgeries (C), vascular (D), visceral surgery (E). GWAS, genome-wide association study.

Figure S8: Genetic correlations between (A) CPSP and other traits. Pink indicates negative correlation, and blue indicates positive correlation. Bar indicates standard error. (B) Surgery subtypes within CPSP. The bottom color scale indicates the range from negative correlation to positive correlation. The number in each square indicates P-value of the correlation.

# Supplementary table legend

Table S1. Selected OPCS4 codes of operations.

Table S2. Chemical name of selected drugs.

Table S3. Case and control numbers in different GWASes. * Ordinal phenotype for time of analgesic use for three months or less (n ≤ 3), between three to six months (3 < n ≤ 6), exceeding six months (n > 6) are 93008, 2030, 893, repectively. † As we use the summary statistics from Marc Parisien et al., the exact case/controls numbers are not available.

Table S4. Lead SNPs passing the suggestive significance level in the GWAS on CPSP development after major or minor surgeries using ordinal chronic postsurgical pain phenotype.

Table S5. Lead SNPs passing the suggestive significance level in the meta-analysis of major, GWAS on CPSP development after minor surgeries and the published CPSP study. The symbol in the direction column: ‘+’ represent positive association, ‘-’ represents negative association, ‘?’ represents not genotyped. The order of symbol in the direction column represent cohorts from: GWAS on CPSP development after major surgeries, GWAS on CPSP development after minor surgeries, hysterectomy cohort (cohort acronym HYS), abodomen and knee cohort (cohort acronym ABKNEE), mastectomy cohort (cohort acronym PMPS), hernia cohort (cohort acronym HRN), knee replacement cohort (cohort acronym TKR), knee arthroplasty cohort (cohort acronym TANK).

Table S6. Functional annotation of candidate SNPs associated with chronic postsurgical pain.

Table S7. Pleiotropic effects of the meta-analysis GWAS lead SNPs in GWAS Atlas.

Table S8. Pleiotropic effects of the meta-analysis GWAS lead SNPs in GWAS catalog.

Table S9. Genes identified by MAGMA gene-based analysis in the meta-analysis of GWAS on CPSP development after major and minor surgeries.

Table S10. Robustness analysis of lead SNPs. The columns headers are the round of iterations. The value in the cell are the P-values in each iteration.

Table S11. Candidate SNPs from the main GWAS examined in the published CPSP Meta-Analysis. We queried 52 candidate SNPs identified from the main GWAS in the published CPSP study. However, some SNPs were not genotyped in any subcohorts of the published CPSP study. In this table, we have excluded SNPs that were not genotyped in any CPSP subcohorts from the published CPSP study.

Table S12. Validation of candidate SNPs reported in the published systematic review.

Table S13. Validation of candidate SNPs reported in previously published CPSP GWASes.

Table S14. Lead SNPs passing the suggestive significance level in the subtype GWAS based on surgery complexity.

Table S15. Lead SNPs passing the suggestive significance level in the subtype GWAS based on surgery sites.

Table S16. Genetic correlations between GWAS on CPSP development after major surgeries and other traits.

Table S17. Genetic correlations between subtype GWASes.

Table S18. Cross check between our CPSP definition with self-reported CPSP in the UKB.

# Reference

1 Bycroft C, Freeman C, Petkova D, et al. The UK Biobank resource with deep phenotyping and genomic data. *Nature* 2018; **562**: 203-9

2 Health A. Schedule of Procedures and/or Fees for Fee Approved Specialists. Available from <https://specialistforms.onlineapps.axahealth.co.uk/>

3 Bulik-Sullivan BK, Loh PR, Finucane HK, et al. LD Score regression distinguishes confounding from polygenicity in genome-wide association studies. *Nat Genet* 2015; **47**: 291-5

4 Auton A, Brooks LD, Durbin RM, et al. A global reference for human genetic variation. *Nature* 2015; **526**: 68-74

5 Jiang L, Zheng Z, Qi T, et al. A resource-efficient tool for mixed model association analysis of large-scale data. *Nat Genet* 2019; **51**: 1749-55

6 Fadista J, Manning AK, Florez JC, Groop L. The (in)famous GWAS P-value threshold revisited and updated for low-frequency variants. *Eur J Hum Genet* 2016; **24**: 1202-5

7 Jia P, Zhan N, Bat BKK, Feng Q, Tsoi KKF. The genetic architecture of blood pressure variability: A genome-wide association study of 9370 participants from UK Biobank. *J Clin Hypertens (Greenwich)* 2022; **24**: 1370-80

8 German CA, Sinsheimer JS, Klimentidis YC, Zhou H, Zhou JJ. Ordered multinomial regression for genetic association analysis of ordinal phenotypes at Biobank scale. *Genet Epidemiol* 2020; **44**: 248-60

9 Parisien M, Reij RRIv, Khoury S, et al. Genome-wide association study suggests a critical contribution of the adaptive immune system to chronic post-surgical pain. *medRxiv* 2023: 2023.01.24.23284520

10 Freidin MB, Tsepilov YA, Palmer M, et al. Insight into the genetic architecture of back pain and its risk factors from a study of 509,000 individuals. *Pain* 2019; **160**: 1361-73

11 Watanabe K, Stringer S, Frei O, et al. A global overview of pleiotropy and genetic architecture in complex traits. *Nat Genet* 2019; **51**: 1339-48

12 Howard DM, Adams MJ, Clarke TK, et al. Genome-wide meta-analysis of depression identifies 102 independent variants and highlights the importance of the prefrontal brain regions. *Nat Neurosci* 2019; **22**: 343-52

13 Pulit SL, Stoneman C, Morris AP, et al. Meta-analysis of genome-wide association studies for body fat distribution in 694 649 individuals of European ancestry. *Hum Mol Genet* 2019; **28**: 166-74

14 Parisien M, van Reij RRI, Khoury S, et al. Genome-wide association studies with experimental validation identify a protective role for B lymphocytes against chronic post-surgical pain. *Br J Anaesth* 2024; **133**: 360-70
