# Supplementary material for: Genome-wide association study on chronic postsurgical pain in the UK Biobank
Source: Br J Anaesth. 2025 Jan 25;134(3):783–92. doi: 10.1016/j.bja.2024.12.008 (PMC11867066; doi:10.1016/j.bja.2024.12.008)
Supplement: Multimedia component 2 [file mmc2.pptx]

## Slide 1
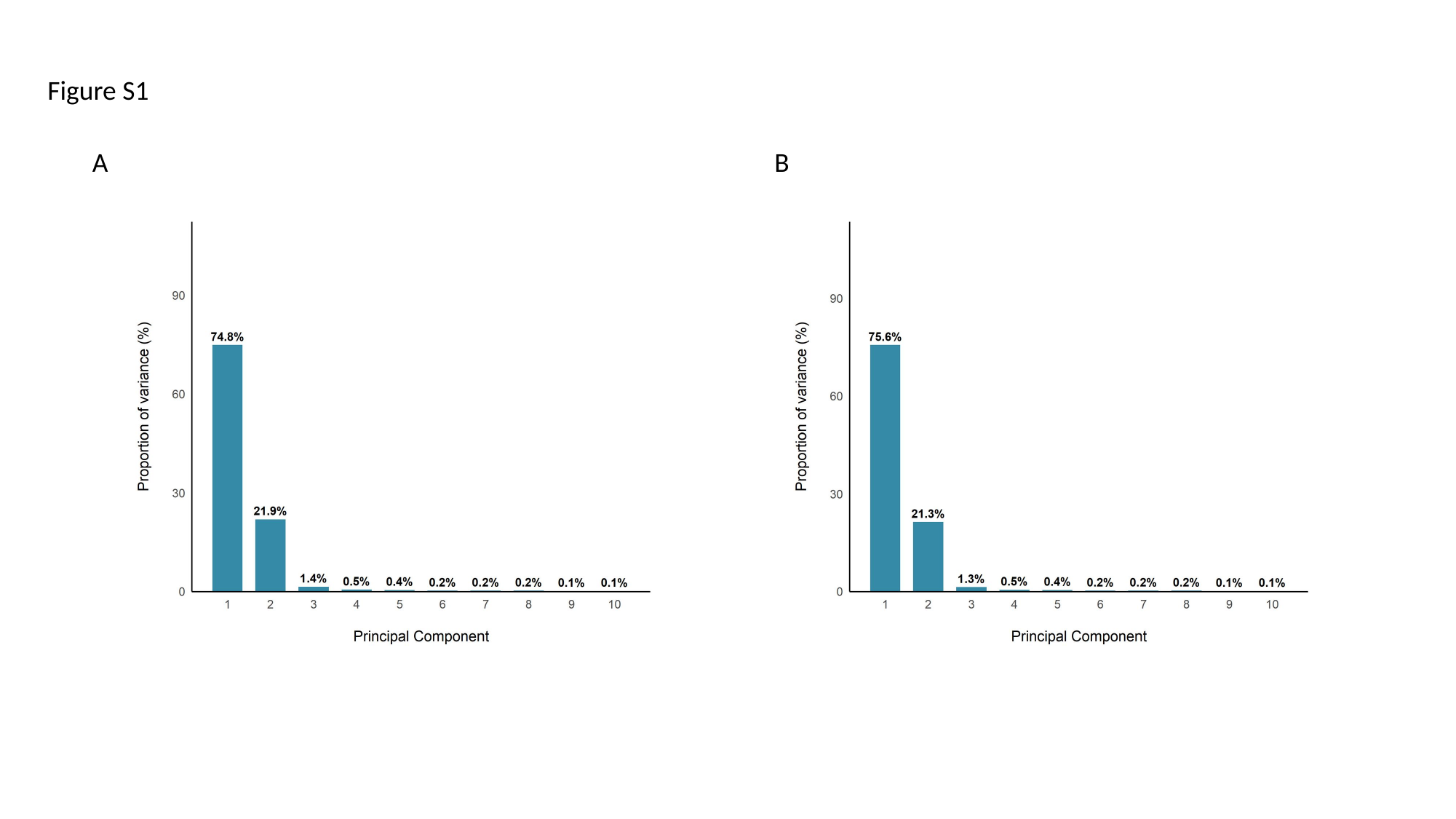

Figure S1
A
B

## Slide 2
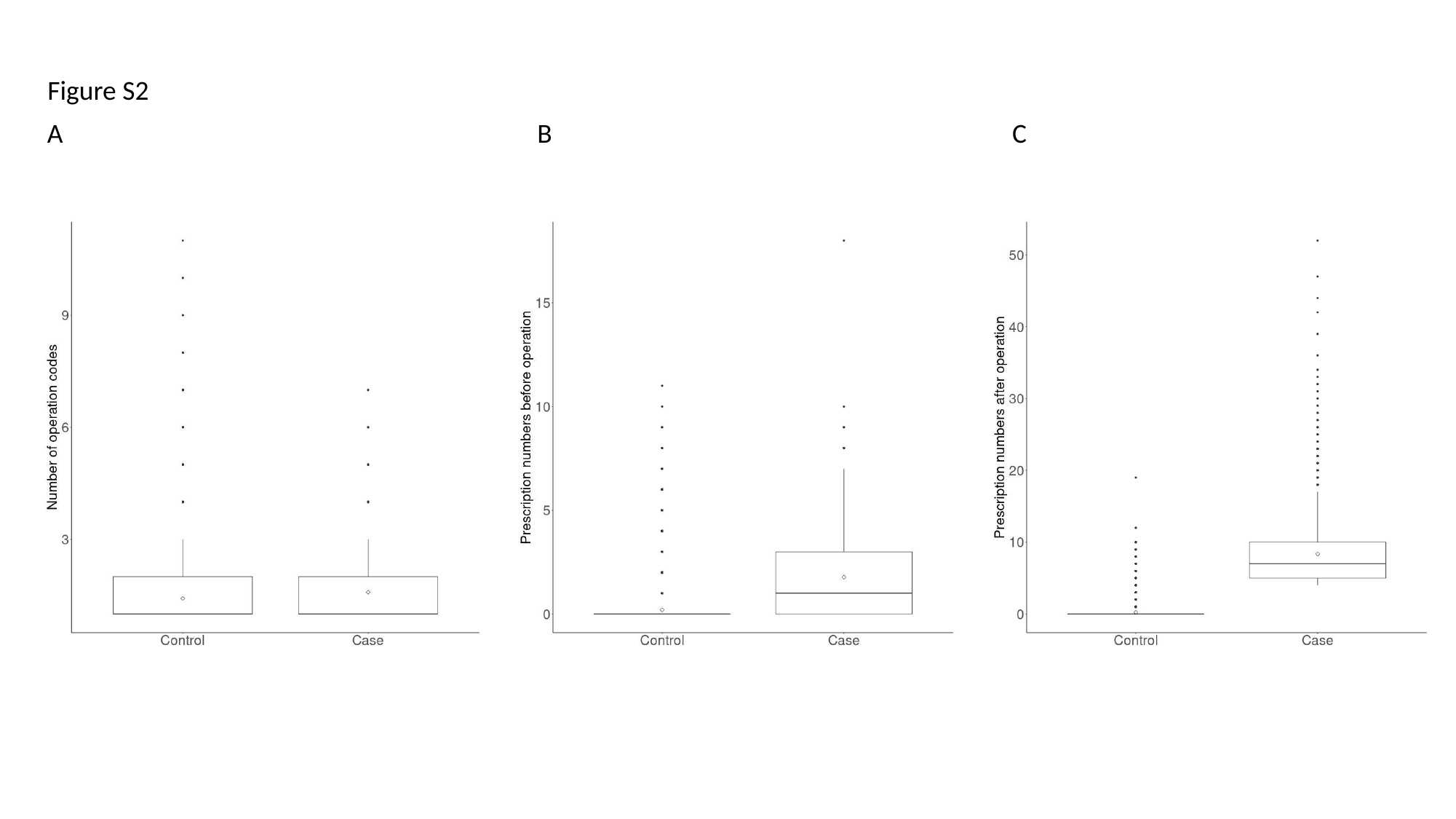

Figure S2
A
B
C

## Slide 3
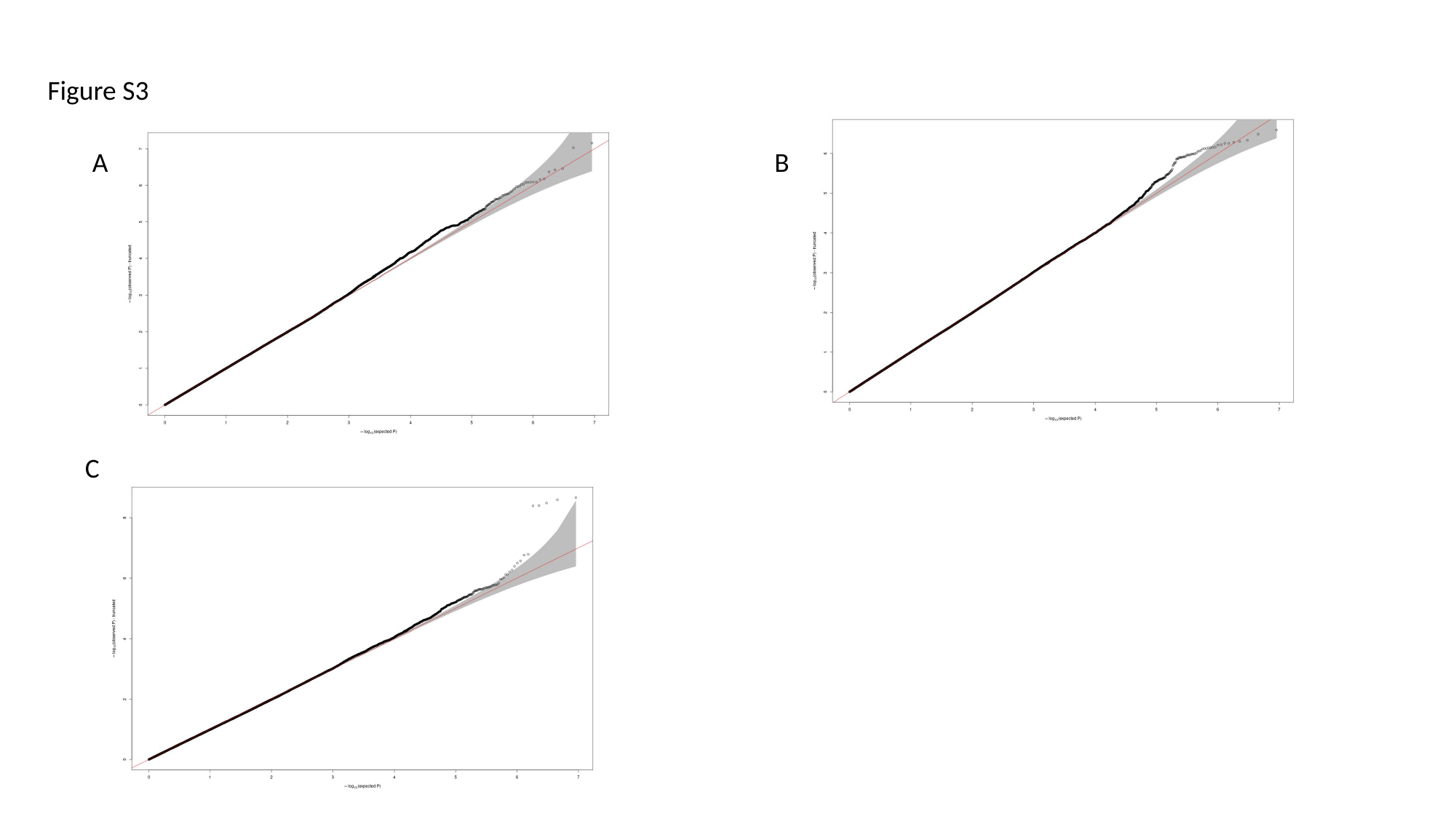

Figure S3
A
B
C

## Slide 4
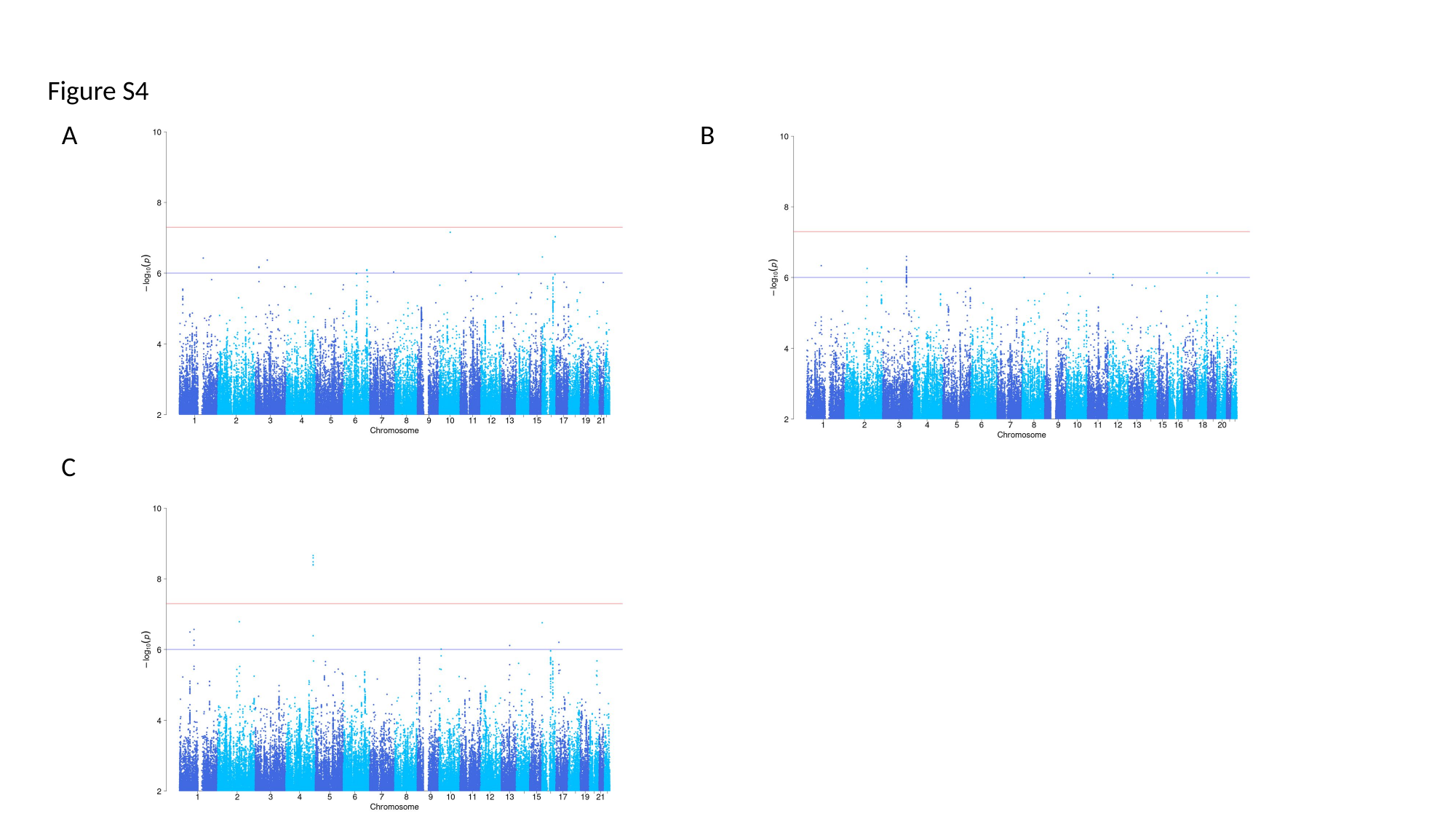

Figure S4
A
B
C

## Slide 5
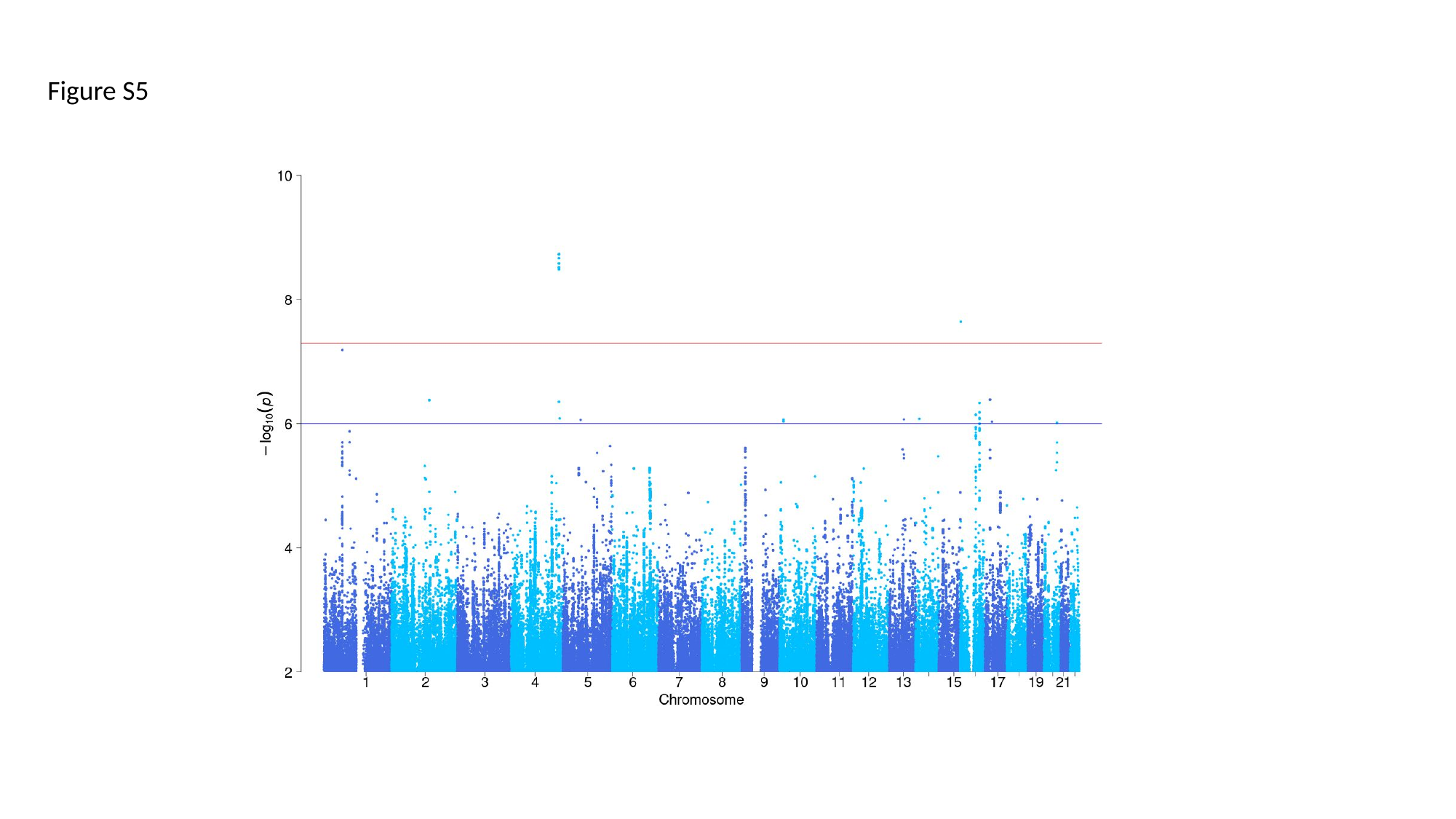

Figure S5

## Slide 6
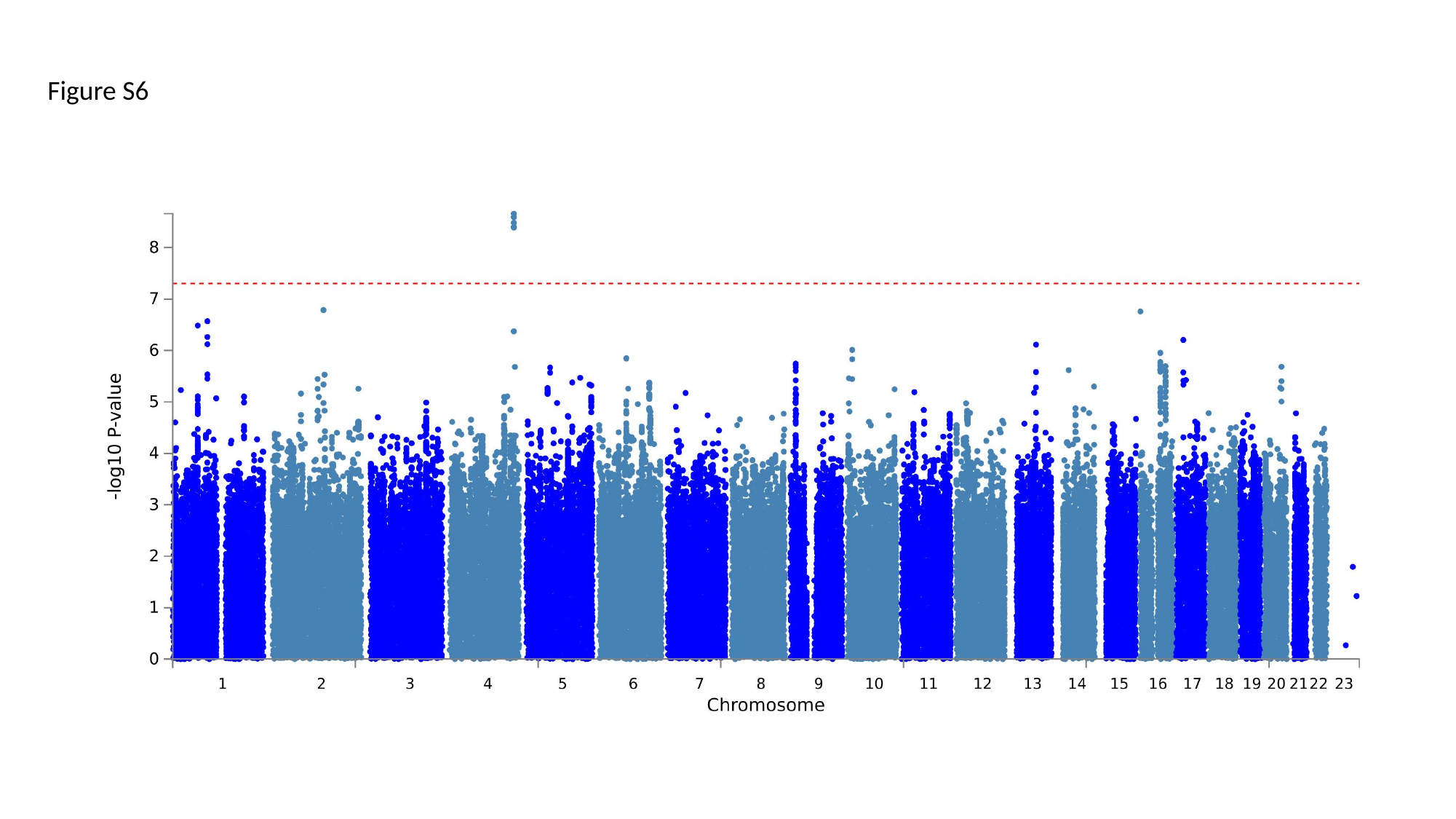

Figure S6

## Slide 7
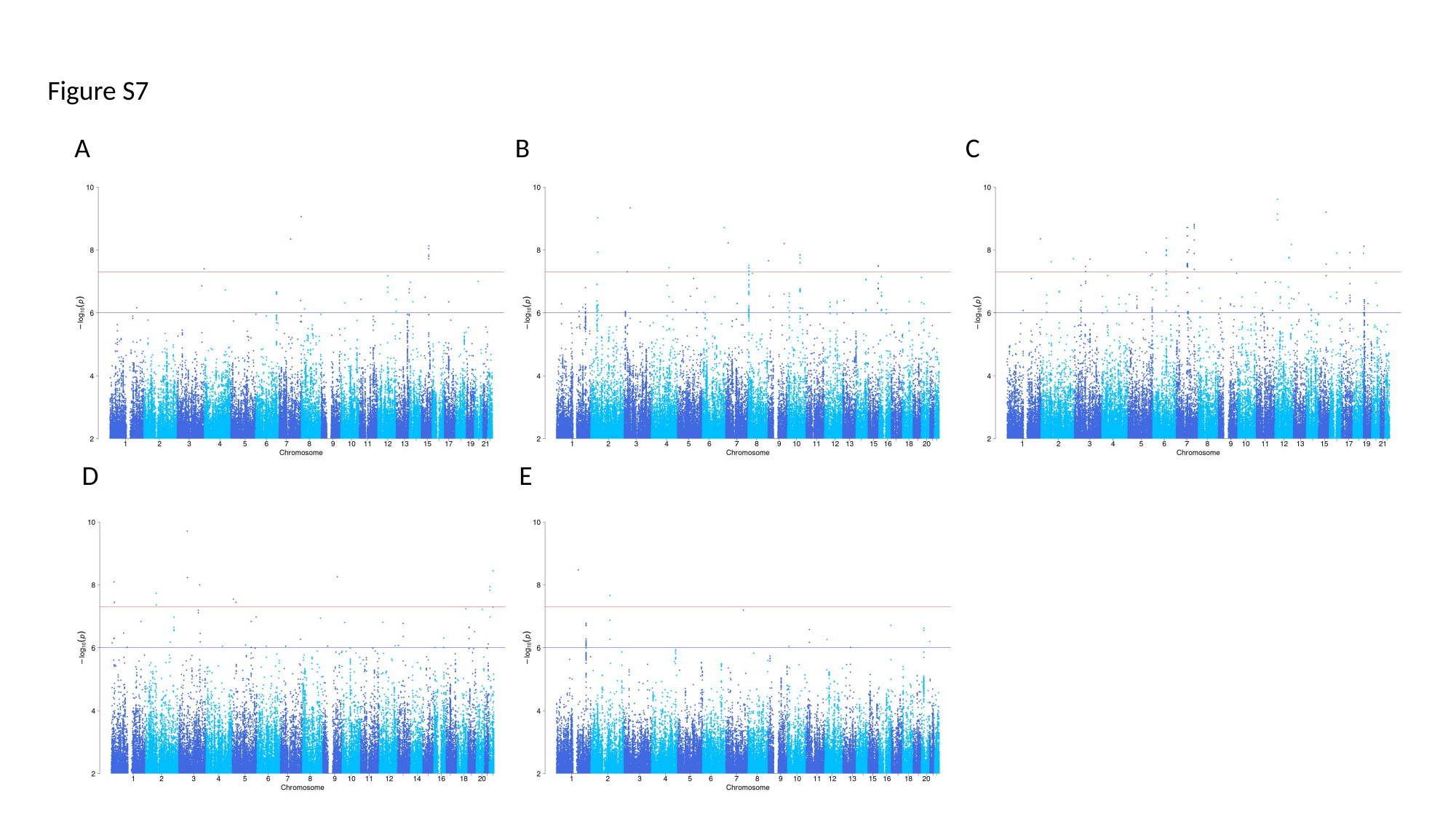

Figure S7
B
C
A
D
E

## Slide 8
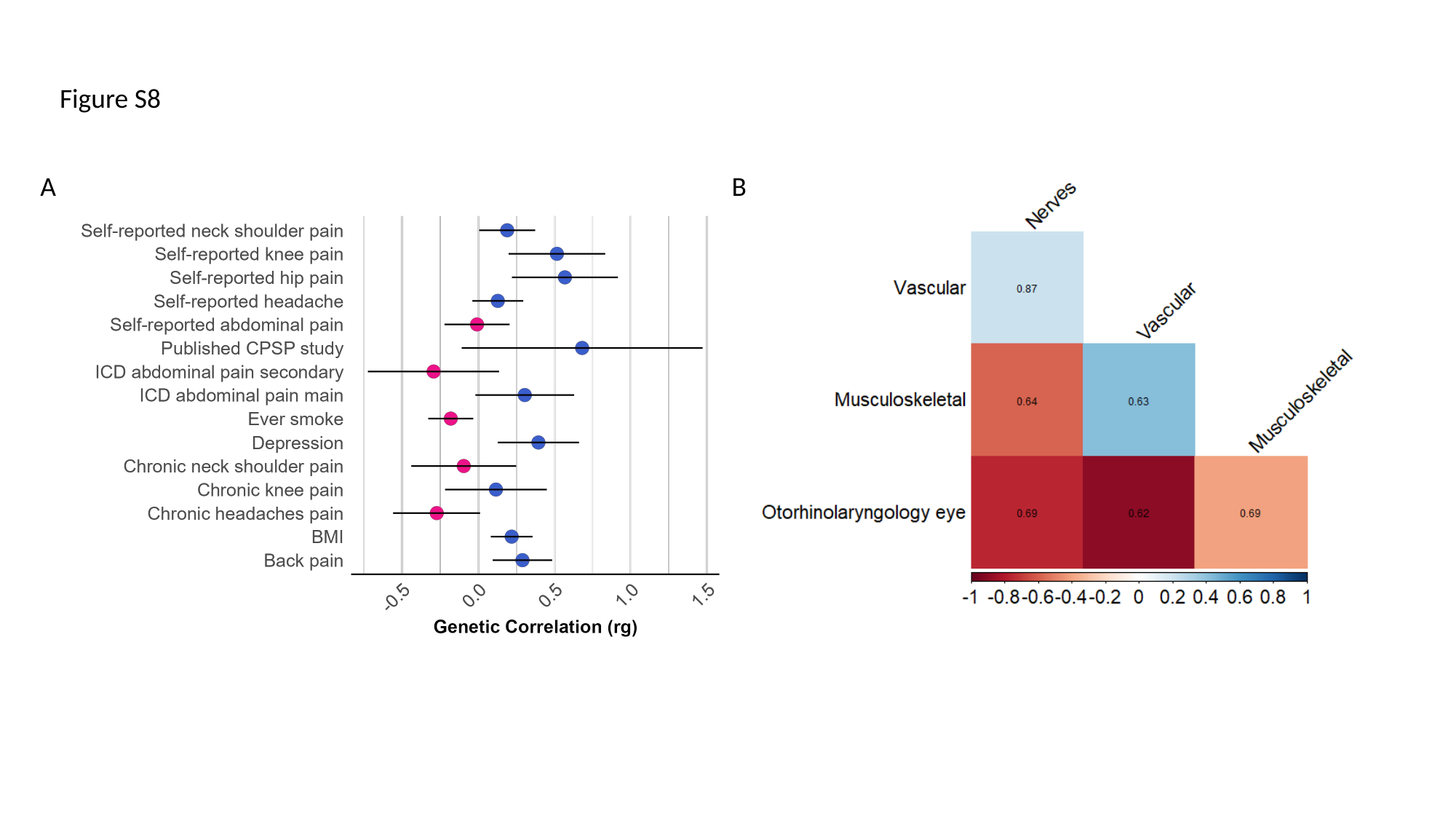

Figure S8
A
B
